# Supplementary material for: Understanding the mechanism of facilitation in hoverfly TSDNs
Source: PLoS Comput Biol. 2025 Oct 13;21(10):e1012986. doi: 10.1371/journal.pcbi.1012986 (PMC12539733; doi:10.1371/journal.pcbi.1012986)
Supplement: Attachment — Submitted filename: Responses to Reviewers.pdf [file pcbi.1012986.s004.pdf]

Dear Editor,

Thank you for your consideration of our paper for publication in PLoS Comp Biol. We have addressed all the reviewer comments as described in detail below, and hope that you now find the paper suitable for publication. The revision includes substantial rewriting of several sections (e.g. Preceding optic flow section, Discussion), a longer Introduction which sets the ecological context better, and a new figure (Fig 11) addressing the questions around latencies, with associated text changes.

Besides the reviewer comments addressed below, we have also added an author summary on the first page of the MS, a CRediT author contributions statement, a list of legends for the supporting information after the references list, and all figures are in .eps format.

The line numbers mentioned in our responses (in black) in this letter reference the revised manuscript (clean copy).

Anindya Ghosh

Reviewers' comments:

Reviewer #1: In this work, Ghosh et al develop computational models of TSDNs in the hoverfly, Eristalis tenax. They test different model variants to elucidate which best represents observed physiological results. The models are derived from interactions between small-field STMDs and wide-field, optic-flow neurons (LPTCs). The manuscript is well written and clear (except for the section on preceding motion). The modelling itself is incremental in its complexity, given that there has been published models exploring interactions between LPTCs and ESTMDs. The parsimonious Circuit 1 would seem to be an obvious solution for the TSDN responses (including facilitation). However, since these interactions have been examined with respect to a different physiological system (TSDN in hoverfly), as well as presenting physiological results, makes this an impactful and novel contribution.

AG: We thank the reviewer for taking the time to review our paper and for providing constructive feedback, which has helped us clarify and improve the paper. We have rewritten the section on preceding optic flow, and now more clearly explain how our models were conceived. This has been described in more detail below.

Comments

Line 18: supressed or facilitated. It would be nice to describe more completely here compared to what? Presumably a target on a blank screen?

AG: We thank the reviewer for mentioning that this was not clear. We have

addressed this by modifying the text to clarify that both the suppression and facilitation are relative to the TSDN response for a target against a homogeneous or stationary background.

Line 27: Models in references 16,17 used a matched temporal filter in addition to strong surround antagonism.

AG: We agree with the reviewer that this needs to be explicitly mentioned, and have corrected the text to reflect this (Ln 51). Please note that other sections of the paragraph have been rewritten to address comments by both Reviewers, and as such, the reference order has also changed.

Line 40: Perhaps reference the ESTMD modelling that included directionality, implemented in a similar way. S. D. Wiederman and D. C. O'Carroll, "Biomimetic target detection: Modeling 2nd order correlation of OFF and ON channels," 2013 IEEE Symposium on Computational Intelligence for Multimedia, Signal and Vision Processing (CIMSIVP), Singapore, 2013, pp. 16-21, doi: 10.1109/CIMSIVP.2013.6583842.

AG: We thank the Reviewer for bringing this important paper to our attention. We imbued directionality to our ESTMDs in a similar way. This is now appropriately referenced in several places in our paper, including in the Introduction to make this clear from the start (Ln 51).

Line 48: Perhaps reference the ESTMD modelling that interacted with LPTC neurons in a similar way. Steven D. Wiederman, Russell S. A. Brinkworth, David C. O'Carroll, "Bio-inspired target detection in natural scenes: optimal thresholds and ego-motion," Proc. SPIE 7035, Biosensing, 70350Z (2008); https://doi.org/10.1117/12.804351 Following this work, there were more labs that modelled such LPTC-ESTMD interactions (see several Brinkworth articles).

AG: We thank the Reviewer for bringing this to our attention. Importantly, while the mentioned references include LPTC input, they were used to *increase* visibility of targets moving against syn-directional clutter. In contrast, hoverfly TSDNs are inhibited under those conditions. Published models can therefore not explain the physiological TSDN responses. We now explain this important distinction and cite the mentioned references in the Introduction (Ln 67), and return to it in the Discussion (Ln 51).

Line 77: Rather than blowfly, should this include citations with Eristalis, which has been an animal model for LPTC investigations (e.g. temporal frequency tuning)

AG: We agree with the reviewer that using *Eristalis* LPTC data could have been appropriate. However, we now explain in the text that we developed models based on spiking neurons, and as *Callipora* H1 and H2 are the best-

studied spiking LPTCs, we used published data for these neurons for model fitting. Importantly, LPTC tuning parameters (velocity, contrast, direction) are similar between *Eristalis* and *Calliphora*, so this choice is unlikely to have any substantial implications on our results. However, it is, as the Reviewer recognizes, an important point, and we therefore now explain this at the start of the Results, please see lines 94-101 onwards.

Line 104: this makes for a dark bar detector. It is the inclusion of strong centre-surround antagonism which creates the target selectivity.

AG: We thank the reviewer for pointing this omission out. We have modified the text to make it clear that the ESTMD uses spatial *and* temporal mechanisms. Please see the modified text on Ln 139 onwards.

Line 120. At the same speed? Or doesn't this matter?

AG: This does matter. Indeed, the strength of inhibition generated by syn-directional background motion follows the velocity tuning of LPTCs [3], adding additional support for LPTC input to the TSDNs. We have modified the text to clarify this important point, and we revisit it in the Discussion. Please see Lines 178 and 452 onwards.

Line 121: As above, the relative speed relationships should be described (if known).

AG: This is a great point raised by the Reviewer, but unfortunately, the facilitation has only been shown in one previous paper [2]. While the paper used different background densities and directions, it did not record responses to different contrasts or velocities, so the text has been left as it was. Please see lines 178-181.

Line 130: "opposite preferred direction" Nice to be clear whether describing the neuron's directionality or the stimulus direction

AG: We thank the reviewer for mentioning that this was unclear. The text has been modified to clarify that "opposite preferred direction" refers to the STMD's directional selectivity, please see line 199.

Line 117: In this section, it is not clear whether the neurons exhibit direction opponency, that is, do they produce below spontaneous levels for motion in the non-preferred direction?

AG: We have added a few sentences to this section (starting on Ln 173) where we introduce TSDNs a bit better. In doing so, we have included information on their direction selectivity, which is an important point for our modelling. As is now mentioned in the text, since TSDNs do not have a spontaneous rate, they do not exhibit classic direction opponency.

Line 136: Wouldn't this be the same for Circuit 2?

AG: We thank the reviewer for mentioning that this was poorly phrased. It would, indeed, be true for all three circuits. We have moved the text to the end of the section and made it applicable to all circuits to reflect this (Ln 216 onwards).

Line 147: In hoverflies, LPTC receptive fields have been mapped with target-like objects, as they induce large responses (presumably non-linear spatial weighting). Therefore, it's not very clear whether star-field is designed as an optic flow or target stimulus, which makes this section difficult to follow.

AG: We thank the reviewer for highlighting that this important point was not clear to the reader. We have modified the second paragraph of this section to make it clear that the starfield background pattern was designed primarily to stimulate optic-flow sensitive neurons, but that they could potentially stimulate different types of neurons due to their specific spatial spatiotemporal structure. This is now discussed on line 187 onwards.

Line 154: fitted to spike rates of peristimulus time histograms?

AG: We did indeed fit this to peri-stimulus time histograms, as suggested by the Reviewer and have added a reference to the Data analysis section in Methods where this is clarified (Ln 698 onwards).

Line 173: The point about spontaneous activity and whether the LPTC models are direction opponent should be made more explicit.

AG: We have modified the text, including referring to Fig 1, to make this point more explicit (Lns 119, 245 onwards), as it is indeed important.

Line 175: What does a "more involved navigation" mean?

AG: This part of the text has been removed in addressing the comment above.

Figure 5: how does pixels/s relate to cm/s

AG: Sideslip at 50 cm/s cannot be directly translated to pixels/s. The starfield pattern simulates self-motion through a space filled with 2 cm spheres. When projected on the 2D screen, these are perspective-corrected to display what the hoverfly would have seen if it were translating at 50 cm/s through this space. Therefore, the speed of each dot on the 2D screen will depend on the simulated distance to the viewer (the hoverfly). This is explained in detail in [1], and briefly in the first paragraph of the 'Comparisons of candidate TSDN circuits' section, as it also relates to a previous point of the Reviewer. We hope that these modifications of the text have sufficiently clarified the issue.

Line 204: "could theoretically". Does it or does it not stimulate STMDs? If so,

what does this mean for the interpretation of results?

AG: We thank the reviewer for asking this. While we have unpublished pilot data suggesting that the starfield background stimulates STMDs, there is no published data verifying this. As such, we err on the side of caution and prefer to keep this cautionary statement. However, we have carefully reworded the section to make this unknown status clearer (Ln 193). The point of the section is that published data made it unclear whether the facilitation came from STMDs or LPTCs, which is why we did new experiments (Fig. 7). We hope that this point is now clear to the Reader.

Liner 211: What is surprising? Is it a discrepancy between prior work? If so, then describe completely so that future research may resolve conflict in the literature.

AG: We apologise for not making this clear. It is indeed surprising considering earlier work using similar backgrounds. We have now edited the paragraph to make this clear, please see line 286 onwards.

Line 222: Is there a rationale for why Circuit 3 should be a possible architecture?

AG: We thank the Reviewer for pointing out that this was not clear. In response to this and earlier comments, we have amended the introductory text under the 'TSDN circuit arrangement' section to clarify why we thought these three circuits were plausible, and what makes the starfield optic flow unique. As now explained in the text, since the fits to the starfield data yielded no obviously preferred circuit, we performed additional electrophysiology experiments using two new backgrounds (cloud and sinusoidal), which allowed us to eliminate circuit 3 as a plausible circuit. We hope that this argument is now clearer in the modified text.

Line 234: overshooting characteristic is not clear

AG: We agree with the reviewer that this was not clear and have amended the text to clarify (Ln 243).

Line 236: if introduced earlier, the concept of target-like objects within forms of background motion would make more sense (rather than a description following)

AG: We thank the Reviewer for pointing out that this was not clear, and for providing constructive suggestions on how to improve the text. In response to this and earlier comments, we have amended the introductory text (first two paragraphs) under the 'TSDN circuit arrangements' section to clarify why we thought these three circuits were plausible, and what makes the starfield optic flow unique. We hope that this argument is now clear in the modified text.

Line 246: how does immediately precede relate to latencies? This entire section

has limited logical flow between undefined physiological data. How long is the preceding stimulus? When are the TSDN responses analysed following? If the rationale is that decay offsets of LPTC neurons affect following TSDN responses, then introduce this concept first.

AG: We apologise for not making this section clear. We have substantially rewritten this section to make the logic clearer, including the timing of stimuli, analysis windows used, how latencies were quantified, etc. In doing this, we have added a figure (now Fig 11) and rearranged the text substantially as suggested by the Reviewer, to clarify our logic. We hope that it is now clearer to the Reader.

Line 250: If TSDN responses are suppressed by preceding optic flow, then doesn't this change the model validation (i.e. that you are building correct models). Given this is the case, it is very important to provide clarity on these attributes, e.g. 'preceding' durations. Moreover, how latency and decay offsets are proposed to underlie model verification. Overall, how latencies of LPTCs and TSDNs relate to both physiological responses (of 'preceding' experiments) as well as model parameters (e.g. low pass filters) is not clear enough (similarly, Figure 10).

AG: As mentioned in the comment immediately above, we have substantially rewritten this section to make the logic more clear, including the timing of stimuli, analysis windows used, how latencies were quantified, etc. In doing this, we have added a figure (now Fig 11) and modified the text substantially to clarify our logic. We hope that it is now clearer how we used this to determine that circuit 1 is the most plausible model.

Figure 9: Do these bar sizes start within the receptive field? If not, how are latencies calculated for stimuli that drift into a neuron's receptive field.

AG: We thank the reviewer for this question. Questions relating to latencies have been addressed in our replies immediately above. The modified text relating directly to target motion relative to the receptive field can now be found on Ln 337, and also see the new Fig 11.

Line 316: The manuscript should explain the spatial weighting of LPTCs observed physiologically and in the modelling. That is, would a hoverfly LPTC respond more strongly to a dot density of 10 dot/m2. Would response be similar above 50 dots/m2?

AG: We thank the Reviewer for highlighting that this was not clear. We have modified the text to clarify how the amount of features in a pattern (dot density) affects the strength of the response of hoverfly LPTCs, and how the dot density affects the response strength of optic flow sensitive descending neurons (see Ln 400 onwards). This text can be found at the start of the section to make this clear from the onset. In addition, we have clarified that LPTC modelling follows

the same dependency on dot density (Ln 406).

Line 329: "plausible combinations" not clear why these architectures were chosen

AG: As mentioned above, we have substantially modified the Introduction to clarify why we thought these three circuits were biologically plausible. The modified text can be found on e.g. Ln 77 onwards.

Line 339: What metric determines whether these results are variable?

AG: Retrospectively, we feel that large parts of the discussion on variability were not valuable to the paper. Therefore, we have removed this part of the Discussion to address other comments by both reviewers.

Line 379: If TSDNs drive foveation, then whether contra or syn directional target and background stimuli covers the visual field of single or both eyes could be very important.

AG: We have rewritten this part of the Discussion in response to this and to Reviewer 2's comments. In particular, we now link back to the Introduction, where we discuss the behavioural relevance of target pursuit in clutter. We also mention that hoverflies have binocular overlap in the frontal visual field, so fronto-dorsal targets would be likely to be seen by both eyes.

Refs – some typo errors

AG: In the References, we have corrected typos that we could see: Ref 32: "2nd" does not have any space between "2" and "nd" now. Ref 58: added spaces in the title when appropriate.

Reviewer #2: Review: Understanding the mechanism of facilitation in hoverfly TSDNs

In this manuscript the authors test 3 different models that could explain the way in which Target Descending Selective Neurons (TSDNs) receive two types of movement inputs: from widefield cells, which carry information about self motion from small target neurons, which carry motion information about a potential prey/mate motion.

The manuscript is well laid out, and the figures are clear. I have a few queries.

AG: We thank the Reviewer for the thoughtful and constructive feedback, which has helped us improve the paper substantially.

1 As far as I can tell, the modelling for self-motion information is based on Reichardt Correlators, as published by Borst et al groups. But there seems to be no mention of more recent publications, for example from Michael Reisers group, showing that the fast excitatory and offset delayed inhibition is what produces the directional sensitivity in flies. This is fundamentally different from the Reichardt Correlator. See Figure 6 in Gruntman et al 2018. I can not understand how such reference is missing in a diptera paper about motion computation. Could the authors use a t4 model instead of Reichardt Correlators? Would this alter the results of the model? Even if the results are not altered, the fact that the Reichardt Correlators is a model now superseded by current evidence should be mentioned and clearly explained.

AG: This is a great point raised by the Reviewer. We have addressed this in several ways. We now mention other, more recent models in the Introduction and Discussion and explain why we chose the EMD for this paper. We believe that using the EMD is appropriate in this context (Ln 470 onwards), as our model LPTC implementation provides a good fit with the biological direction and temporal frequency tuning data across the range of temporal frequencies used in our subsequent experiments in both the PD and ND, see Fig 1. In addition, the response onset of our model is similar to biology, across temporal frequencies (Fig 2). The model also recapitulates the asymmetry in PD vs ND motion responses well (Fig 1). The main difference between the Gruntman model and the EMD that could affect our results is the asymmetry in PD vs ND responses, caused by the spatially offset delayed inhibition (Fig 5 in the mentioned Gruntman paper). However, at the higher background velocities (90 degrees/s) used in our subsequent experiments, PD and ND motion show a similar time course, so it is unlikely to affect our conclusions. This important point is now addressed in the Discussion.

2. In the intro, there seems to be no mention about the real world importance of both types of information getting to the TSDNs. This is only mentioned at the very end of the conclusion, but I think that to ground the reader, it should be included in the introduction. This also sets the stage for explaining why

the same motion for target and background results in minimal TSDN activity (the target didn't move, the pursuer did), or why the background moving in the opposite direction to the target should facilitate the TSDN activity (the target is moving faster the pursuer…and it is therefore slipping away). the biological system needs to compare both signals (is the target motion detected at the retina level caused by self-motion, or is the target moving in a way that differs from the predator). Comparing the two signals gives the true target motion that needs to be corrected, and indeed is the signal necessary for a proportional navigation system which explains predation in other diptera. This computation needs to be fast and accurate: which would push the system to be robust and minimal number of computation steps. This would all argue for the 1st model to be the most likely one.

AG: This is a great point. We have now modified the start of the Introduction to discuss the behavioural significance of the background modulation of TSDN responses. This can be found on Ln 22 onwards. In addition, we return to this important point in a modified Discussion, mentioning how the simple model helps in making decisions fast and accurate, with reference to other predatory diperans with much better described behaviors (Ln 501 onwards).

3. I must be missing something when it comes to the 1st part of the results: Model 3 is a complex model made to fit the very specific conditions in which the data was collected,. It is not surprising that it was the best performing model for that data set and that it does not fit anything else well. At that point in the paper, this set off alarm bells for the reader: Do the authors not understand that they have artificially created a fit? It is great that the authors went ahead and collected more data to show that this was indeed the case, but maybe an acknowledgement that Model 3 was expected to be the best performing because it design to fit the specific circumstances of that data set, would warn the readers that the authors were aware of these pitfalls.

AG: We thank the Reviewer for highlighting that our argument was not clear. We have now modified the text to clarify that we started out with three plausible circuits, based on our 2021 PNAS paper [2]. The PNAS paper used a starfield stimulus consisting of a multitude of targets, which could theoretically stimulate both LPTCs and STMDS. Each of the three models was equally plausible at that point, as we now explain in the text. When, in this paper, we fitted the three models to the published data, we still could not distinguish between them, as they performed almost equally well. We therefore did new experiments with backgrounds that can only stimulate LPTCs (e.g. the sinusoidal grating), which allowed us to rule out circuit 3.

We have modified the text in several places to make this strategy clearer to the reader. The modified text can be found in several places, primarily in the 'TSDN circuit arrangements' section, Ln 186 onwards.

4. the authors mention that TSDNs are downstream of the other target neurons. Do we KNOW that? Or is it believed that this is the case?

AG: This is a great point. We do not KNOW that TSDNs are immediately post-synaptic, or even downstream of STMDs. However, there are several lines of indirect evidence to strongly support this. We have modified the text at the start of the 'TSDN circuit arrangements' section to specify the evidence available to support this notion, see Ln 171 onwards.

5. The authors mention the variability of the TSDN responses, but fail to mention that TSDNs are not a homogeneous population. Much of the paper is based on dragonfly data, but dragonfly TSDNS have clearly different roles, in addition to target selectivity, some of them are looming sensitive and others carry predictive information. What type of TSDNs did the authors record from? Do they know it was always the same type?

AG: While dragonfly TSDNs constitute a heterogeneous population of neurons, the TSDNs that we record from in hoverflies are surprisingly homogeneous [4]. Therefore, we do not believe that the variability is caused by recording from different types of TSDNs. We have added some additional text to the Results section (under 'TSDN circuit arrangements'), introducing the TSDNs better in response to this and other comments. In addition, we have added text to the Discussion to clarify this important point.

Line 15. We don't know that they are postsynaptic partners right?

AG: We apologise for not clarifying this. We now say that they are presumed post-synaptic in the Introduction, and have added a section further down to include the evidence, as addressed in an earlier response.

Line 76. The model fails to capture the width of the response, with the biological one being wider. This doesn't seem addressed. Could the authors please explain the reason behind this discrepancy?

AG: We thank the reviewer for raising this question. The width of the velocity tuning varies between LPTCs and conditions. In modelling, the shape can be modified by changing the filters in the model. The text in this section has been restructured to address this (Ln 116).

Lines 87-97 – the jump between those two paragraphs is difficult for a reader to follow. A rewrite may help.

AG: We agree with the Reviewer that this section was sub-optimal. We have reorganised the text to improve the flow (Ln 145 onwards).

Figure 3: here the biological curve is wider again. Why is that?

AG: We have modified the text on Ln 162 to clarify that we aimed to mimic the published ESTMD model, more than the biology. However, there is a close match with biology at the heights and speeds used in our subsequent experiments. This is now addressed in the text.

Line 168 ' the STMD output remains unchanged'. Is this model or biological data.? This isn't made clear to the reader. Same for label of Figure 5 blue line.

AG: We have clarified this important point in the text where we refer to Fig 5, including clear references to the blue/cyan STMD data. In addition, we have modified Fig 5's caption so that it is hopefully clearer.

Figure 7: D: there are more spikes for this treatment, but the DC line seems to go lower. Is there a sustained inhibition then? This seems to occur in other treatments but it is not addressed?

AG: This is a great observation. However, as our recordings are extracellular, we are not confident in making any conclusions based on any potential DC offsets, just like we would not make a point about the action potential amplitude in an extracellular recording. To do any proper analysis of this, and to verify that there is a real DC offset, we would need to do intracellular recordings, which is beyond the scope of this paper.

Figure 7: 3 different TSDNs. Which TSDNs? Did they have the same properties?

AG: This is an important point, which was not clear in the previous version of the paper. As mentioned above, while dragonfly TSDNs constitute a heterogeneous population of neurons, the TSDNs that we record in hoverflies are surprisingly homogeneous [4]. We have added text to the Results section (under 'TSDN circuit arrangement') to clarify this, together with why we think that TSDNs are post-synaptic to STMD. We also address this issue in the Discussion. We hope that this has now clarified that the three neurons would have similar properties.

Line 366. This section appears to belong in Results, not discussion? Also, why are the results not shown? They should always be included somewhere.

AG: As mentioned above, in rewriting the Discussion as part of requests by both Reviewers, we felt that this part was not needed anymore. As such, this part of the Discussion has been removed.

Q on methods: As mentioned by the authors TSDNs are not rate neurons. The authors use a convolution method to turn the data into Spike Density Function. But they give no reference for this method. This is important for the reader that wants to know how appropriate it is...etc For example,is it a fixed kernel? Please provide a reference.

AG: We thank the reviewer for mentioning this. We have added ref. [5] to address this (Ln 686).

References

[1] Richard Leibbrandt, Sarah Nicholas, and Karin Nordström. “The impulse response of optic flow-sensitive descending neurons to roll m-sequences”. In: *Journal of Experimental Biology* 224.23 (2021), jeb242833.

[2] Sarah Nicholas and Karin Nordström. “Facilitation of neural responses to targets moving against optic flow”. In: *Proceedings of the National Academy of Sciences* 118.38 (2021), e2024966118.

[3] Sarah Nicholas et al. “Integration of small-and wide-field visual features in target-selective descending neurons of both predatory and nonpredatory dipterans”. In: *Journal of Neuroscience* 38.50 (2018), pp. 10725–10733.

[4] Yuri Ogawa et al. “Neural responses to reconstructed target pursuits”. In: *bioRxiv* (2023), pp. 2023–05.

[5] Attila Szűcs. “Applications of the spike density function in analysis of neuronal firing patterns”. In: *Journal of neuroscience methods* 81.1-2 (1998), pp. 159–167.
