# Supplementary material for: Understanding the mechanism of facilitation in hoverfly TSDNs
Source: PLoS Comput Biol. 2025 Oct 13;21(10):e1012986. doi: 10.1371/journal.pcbi.1012986 (PMC12539733; doi:10.1371/journal.pcbi.1012986)
Supplement: Attachment — Submitted filename: Responses to Reviewers2.pdf [file pcbi.1012986.s005.pdf]

Dear Editor,

Thank you for your consideration of our paper for publication in PLoS Comp Biol. We have addressed all your comments as described below, and hope that you now find the paper suitable for publication. The revision includes the text changes requested by the editor.

With regards,
Anindya Ghosh

Comments

It is helpful to have added mention and some discussion of the T4/T5 models but their introduction could be clearer: these are not just 'other recent' models but models far more strongly justified by biological evidence than the preceding phenomenological models.

AG: We thank the Editor for mentioning that this could be clearer. We have addressed this by modifying the text to reflect that the T4/T5 models are more biophysically grounded than the previous phenomenological models.

In the author summary, the problem is described as 'computationally hard' but nothing in the text explains why it is hard (indeed it seems a simple model is sufficient). Both the summary and abstract imply the solution here could be of relevance to bio-inspired robotics but this receives no further mention anywhere in the paper. Either add some relevant discussion about how the equivalent problem is currently addressed in robotics and/or computer vision, or drop these references to robotics.

AG: We thank the Editor for highlighting this. We have removed the phrase 'computationally hard' from the author summary, as well as references to how the model could be applied to robotics from the author summary and abstract.
